# Clodronate-liposome-mediated depletion of tumour-associated macrophages: a new and highly effective antiangiogenic therapy approach

**DOI:** 10.1038/sj.bjc.6603240

**Published:** 2006-07-11

**Authors:** S M Zeisberger, B Odermatt, C Marty, A H M Zehnder-Fjällman, K Ballmer-Hofer, R A Schwendener

**Affiliations:** 1 Molecular Cell Biology, Laboratory of Biomolecular Research, Paul Scherrer Institute, CH-5232 Villigen-PSI, Switzerland; 2 Department of Pathology, University Hospital, CH-8091 Zürich, Switzerland

**Keywords:** clodronate, liposomes, tumour-associated macrophages, macrophage depletion, antiangiogenic tumour therapy

## Abstract

Tumour-associated macrophages, TAMs, play a pivotal role in tumour growth and metastasis by promoting tumour angiogenesis. Treatment with clodronate encapsulated in liposomes (clodrolip) efficiently depleted these phagocytic cells in the murine F9 teratocarcinoma and human A673 rhabdomyosarcoma mouse tumour models resulting in significant inhibition of tumour growth ranging from 75 to >92%, depending on therapy and schedule. Tumour inhibition was accompanied by a drastic reduction in blood vessel density in the tumour tissue. Vascular endothelial growth factor (VEGF) is one of the major inducers of tumour angiogenesis and is also required for macrophage recruitment. The strongest effects were observed with the combination therapy of clodrolip and a VEGF-neutralising antibody, whereas free clodronate was not significantly active. Immunohistologic evaluation of the tumours showed significant depletion of F4/80^+^ and MOMA-1^+^ and a less pronounced depletion of CD11b^+^ TAMs. Blood vessel staining (CD31) and quantification of the vessels as well as TAMs and tumour-associated dendritic cells (TADCs) in the A673 model showed reduction rates of 85 to >94%, even 9 days after the end of therapy. In addition, CD11c^+^ TADCs, which have been shown to potentially differentiate into endothelial-like cells upon stimulation by tumour released growth and differentiation factors, were similarly reduced by clodrolip or antibody treatment. These results validate clodrolip therapy in combination with angiogenesis inhibitors as a promising novel strategy for an indirect cancer therapy aimed at the haematopoietic precursor cells that stimulate tumour growth and dissemination and as a tool to study the role of macrophages and dendritic cells in tumorigenesis.

Solid tumours are not only composed of malignant cells, but are complex organ-like structures comprising many cell types, including a wide variety of migratory haematopoietic and resident stromal cells (Pollard, 2004). The role of leucocyte infiltration into solid tumours was noticed more than a decade ago. Migration of these cell types into tumours has been interpreted as evidence for an immunological response of the host against a growing tumour, but more recently it became clear that tumours are largely recognised as self and lack strong antigens. Instead, they appear to have been selected to manipulate the host immune system to prevent rejection and to use it to facilitate their own growth and spread (Khong and Restifo, 2002; Ochsenbein, 2005). This led to the proposal that haematopoietic cell infiltrates composed of myeloid cells, neutrophils, dendritic cells (DCs), eosinophils, mast cells, lymphocytes and macrophages have a causal role in carcinogenesis. Clinical data collected from a wide range of solid tumours underscore these findings given that high densities of leucocytic infiltrations, most notably macrophages, correlate with poor prognosis of the diseases (Chen *et al*, 2005; Joyce, 2005).

Tumour-associated macrophages (TAMs) produce a vast number of factors that promote tumorigenesis. The most prominent include basic fibroblast growth factor (bFGF), vascular endothelial growth factor (VEGF), platelet-derived growth factor (PDGF), transforming growth factor beta (TGF*β*), the angiopoietins (Ang1 and Ang2), interleukins such as IL-1 and IL-8, tumour necrosis factor-*α* (TNF-*α*), thymidine phosphorylase (TP), the matrix metalloproteinases MMP-9 and MMP-2, nitric oxide (NO) and chemokines (Pollard, 2004). The coordinated spatial and temporal expression of these molecules results in proliferation and migration of endothelial cells (ECs), remodelling of the extracellular matrix and formation of stabilised blood vessels. Macrophages are perfectly apt to promote these processes, as their monocytic precursors migrate to specific locations such as hypoxic tumour tissues (Mantovani *et al*, 2004; Murdoch *et al*, 2004; Pollard, 2004), where they differentiate and synthesise angiogenic molecules. It is well established that tumours require angiogenesis to grow beyond a size of a few millimetres. Angiogenesis has also been found to be crucial for extensive tumour growth and metastasis by providing oxygen and nutrients and removal of waste products (Folkman, 2003). The VEGF family of growth factors, consisting of structurally highly related proteins and their corresponding receptors, play an essential role in angiogenic processes in haematological malignancies and in solid tumours (Podar and Anderson, 2005). Several therapeutic approaches targeting VEGF or its receptors show good clinical results (Joyce, 2005; Vosseler *et al*, 2005). Furthermore, recent studies indicate that vasculogenesis, mediated by the recruitment of bone-marrow-derived vascular leucocytes, which simultaneously express both endothelial and dendritic cell markers and differentiate into endothelial-like cells, plays an important role in tumour angiogenesis (Coukos *et al*, 2005). The crucial role macrophages play in pathological lymphangiogenesis was documented in a recent publication by Maruyama *et al* (2005), who provided evidence that CD11b^+^ macrophages are able to transdifferentiate into lymphatic endothelial cell clusters that join existing lymph vessels in a mouse corneal transplantation model.

Bisphosphonates are compounds used in the clinic to prevent or inhibit the development of bone metastases or excessive bone resorption and for the therapy of inflammatory diseases such as rheumatoid arthritis and osteoarthritis (Rogers *et al*, 2000; Ross *et al*, 2004). Recently, the use of bisphosphonates as antiangiogenic agents has been found to suppress solid tumour growth and metastases (Giraudo *et al*, 2004). With the encapsulation of the bisphosphonate clodronate into liposomes (clodrolip), an efficient reagent for the selective depletion of macrophages has been developed and successfully applied in several immunological studies (Seiler *et al*, 1997; Tyner *et al*, 2005). We therefore investigated the possibility whether depletion of TAMs would inhibit tumour angiogenesis and consequently tumour growth and dissemination. Here, we show for the first time that clodronate-liposome-mediated TAM depletion inhibits tumour growth, presumably through blocking of tumour angiogenesis. In our experiments, tumour-bearing mice were treated with clodrolip as single therapy in comparison to free clodronate and in combination with anti-VEGF single chain fragment antibodies (Abs), resulting in drastic tumour growth inhibition and exhaustion of TAM and TADC cell populations.

## MATERIALS AND METHODS

### Cells and mice

Murine F9 teratocarcinoma (CRL-1720), human A673 rhabdomyosarcoma (CRL-1598) were from American Type Culture Collection (ATCC) and HUVE cells from PromoCell (Heidelberg, Germany). The cell culture media DMEM and HUVEC growth medium were from Gibco, Paisley, UK. Peritoneal macrophages were freshly isolated from Sv129 mice. Female Sv129 mice and CD1 nude mice were from Charles River Wiga (Sulzfeld, Germany) and kept in standard housing and normal diet at the animal facility of the Paul Scherrer Institute. Animal studies were approved by the Veterinary Department of the Canton Aargau, Switzerland and performed under the licenses (No. 75533 and 75534) issued to RA Schwendener. The ethical guidelines that were followed meet the standards required by the UKCCCR guidelines (Workman *et al*, 1998).

### Clodronate liposomes

Clodronate liposomes, termed clodrolip, were essentially prepared as described before (Seiler *et al*, 1997). Briefly, for the preparation of 40 ml of clodronate liposomes, the following composition was used. Soy phosphatidylcholine (4.0 g, Epikuron 200, Lukas Meyer, Hamburg, Germany), cholesterol (0.6 g, Fluka, Buchs, Switzerland) and D,L-*α*-tocopherol (0.02 g, Merck, Darmstadt, Germany) corresponding to 1 : 0.3 : 0.01 mol parts were prepared by freeze–thawing and filter extrusion. The dry lipid mixture was solubilised in a physiologic phosphate buffer (20 mM, pH 7.4) supplemented with mannitol (230 mM) and 2.64 g clodronate (clodronic acid disodium salt tetrahydrate, CH_2_Cl_2_Na_2_O_6_P_2_ × 4 H_2_O, Bioindustria LIM, Novi Ligure, Italy). The resulting multilamellar vesicles were freeze–thawed in three cycles of liquid nitrogen and water at 40°C, followed by repetitive (5–10 ×) filter extrusion through 400 nm membranes (Nuclepore, Sterico, Dietikon, Switzerland) using a Lipex™ extruder (Lipex Biomembranes, Inc., Vancouver, Canada). Nonencapsulated clodronate was removed by dialysis (Spectrapore tube, 12–14 000 mol.wt. cutoff). Liposome size and homogeneity were routinely measured with a Nicomp laser light scattering particle sizer (Nicomp 370, Sta. Barbara, CA, USA). Routinely prepared small unilamellar clodrolip liposomes contain approximately 20 mg clodronate ml^−1^ and have a mean diameter of 135 ± 55 nm.

### Cytotoxicity assay

The *in vitro* cytotoxicity of clodronate was assessed as described before (Marty *et al*, 2004). Briefly, cells were incubated in 96-well plates with liposomes, clodronate and clodrolip (6 h, 37°C, 1 mg clodronate ml^−1^) and cell viability was determined by addition of WST-1 reagent (Roche Diagnostics, Mannheim, Germany) according to the manufacturer's recommendations.

### Tumour models and therapies

Exponentially growing F9 teratocarcinoma (7 × 10^6^ 50 *μ*l^−1^) or A673 rhabdomyosarcoma cells (6–8 × 10^6^ 50 *μ*l^−1^ mixed 1 : 1, v v^−1^ with Matrigel, Beckton Dickinson, Basel, Switzerland) were injected subcoutanously (s.c.) on the flanks of mice. Treatment was started 6 h after inoculation of F9 cells (female Sv129 mice) and 24 h after inoculation of A673 cells (female CD-1 nude mice), respectively. The mice (6–8/group) received clodronate dissolved in phosphate buffer (PB, 67 mM, pH 7.4) or clodrolip by intraperitoneal (i.p.) injection as initial dose of 2 mg 20 g^−1^ mouse body weight, followed by 1 mg for the subsequent doses. The Abs were given at 0.5 mg 20 g^−1^ mouse body weight in 100 *μ*l PB by intravenous (i.v.) injection into the tail vein. Tumour growth was measured in a blinded fashion with a caliper and volumes were calculated using the equation: *V*=*πab*^2^/6 (*a*=largest tumour diameter, *b*=perpendicular diameter). Relative percentual tumour growth was normalised to day one. Mice were killed 8–22 days after onset of treatment and tumours and spleens removed for histology.

### Immunohistochemistry

Histological and immunohistochemical (IHC) assays were carried out as described previously (Mrkic *et al*, 2000). Briefly, tissue specimens for IHC analysis were collected and snap frozen. For staining of cell differentiation markers, the following primary rat or rabbit anti-mouse mAbs were used: CD11b (M1/70), CD11c (HL3), CD31 (PECAM-1), FDC (rat-anti-mouse FDC), B220 (RA3–6B2, all PharMingen, San Diego, CA, USA), CD68 (rat-anti-mouse CD68, Serotec), F4/80 (A3–1, ATCC), CD3 (KT3, ATTC), MOMA1 and ER-TR9 (BMA Biomedicals, Augst, Switzerland) and LYVE-1 (ReliaTech, Braunschweig, Germany). Primary Abs were detected by sequential incubation with alkaline phosphatase-labelled species-specific secondary Abs. Alkaline phosphatase was visualised using naphthol AS-BI (6-bromo-2-hydroxy-3-naphtholic acid-2-methoxy anilide) phosphate and new fuchsin as a substrate, yielding a red reaction product. Cell nuclei were counterstained with hemalum.

### Anti-VEGF antibodies

Recombinant cfVEGF_164_ (*Canis familiaris*) and VEGF-E (originating from the orf-family of pox viruses) were produced in *Pichia pastoris* and purified by affinity chromatography as described previously (Scheidegger *et al*, 1999). Recombinant hVEGF_165_ was kindly provided by A Zisch (Institute of Obstetrics, University Hospital Zürich, Switzerland). Recombinant mVEGF_164_ (purity >95%) was from ReliaTech. The synthetic ETH-2 human scFv antibody phage library was from D Neri (Institute of Pharmaceutical Sciences, ETH Zürich, Switzerland) (Pini *et al*, 1998). For the selection of species crossreactive scFv Abs from the phage library, three rounds of panning with recombinant mVEGF_164_ and hVEGF_165_ were performed. Immunotubes (Maxisorp, Nunc) were coated with 25 *μ*g ml^−1^ mVEGF_164_ in PB (first and second panning) followed by hVEGF_165_ (third panning) for 12 h at room temperature. Selected scFv antibodies were screened by ELISA for binding to recombinant hVEGF_165_ and mVEGF_164_ using the M2 anti-FLAG-antibody (Sigma, Buchs, Switzerland) for detection. The formation of (scFv′)_2_ Ab dimers was accomplished by introduction of C-terminal cysteines as described (Marty *et al*, 2002). After subcloning into the yeast expression vector pPICZ*α*A, the selected homodimeric Abs (SZH9, V65) (Vitaliti *et al*, 2000) and the unspecific control Ab A1 were produced and purified as described (Marty *et al*, 2002). Binding constants against mVEGF_164_ and cfVEGF_164_ of SZH9 were determined with a competitive ELISA assay (Friguet *et al*, 1985). For the determination of pharmacokinetic parameters, SZH9 was iodinated (^125^I) using the Iodogen method. Female CD1 nude mice bearing subcutaneously implanted A673 cells (6–8 × 10^6^) at each flank were injected with 3 *μ*g (1.85 × 10^5^ MBq) scFv Abs in 0.1 ml phosphate buffer (PB, 67 mM, pH 7.4). Mice (three/group) were killed at 0.17, 0.5, 1, 2, 4, 6 and 24 h after injection. Antibody concentrations in blood, liver, kidneys and tumours were determined and expressed as percent of injected dose per gram tissue (%ID g^−1^). Pharmacokinetic parameters were calculated with GraphPad Prism 4 software.

### Statistical analysis

All measurements are shown as average±s.e.m. Statistical analysis of data from growth inhibition experiments was performed using a two-tailed, unpaired *t*-test. All *P-*values were calculated *vs* PB controls unless indicated otherwise. *P*-values of <0.05 were considered as statistically significant. The quantification of the IHC data was performed with the image analysis software analySIS (Soft Imaging System, Münster, Germany).

## RESULTS

### Selective cytotoxicity of clodrolip

**Figure 1:**
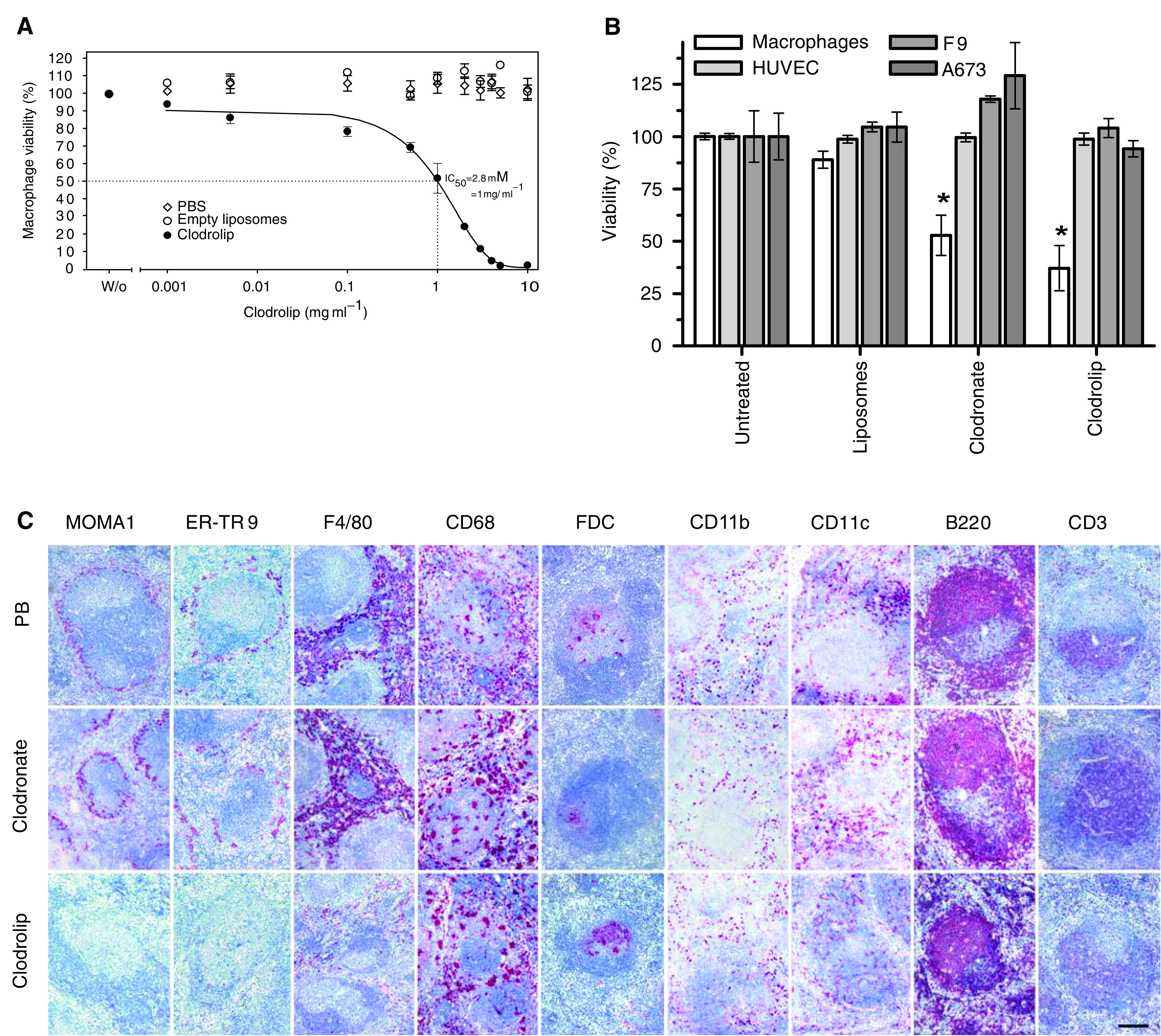
*In vitro* and *in vivo* effects of free and liposome encapsulated clodronate (clodrolip). (**A**) Concentration-dependent cytotoxicity of clodrolip on macrophages (isolated from Sv129 mice by peritoneal lavage) *in vitro*. (**B**) Cytotoxicity of clodronate or clodrolip on different cells *in vitro*. Macrophages, HUVE, F9 and A673 cells were cultured in the presence of 1 mg ml^−1^ clodronate or clodrolip for 6 h. Results are means±s.e.m. (*n*=3). Statistical analysis: ^*^P<0.05 *vs* untreated cells. (**C**) Selective depletion of spleen cell populations after treatment with clodronate and clodrolip. Spleen tissues obtained from immunocompetent Sv129 mice injected with PB, clodronate or with clodrolip are shown (initial dose 2 mg 20 g^−1^ mouse body weight, followed by 1 mg, every 4 days, i.p.). Spleens were removed and sections IHC stained for marginal zone metallophilic MOMA1^+^, marginal zone ER-TR 9^+^, red pulp F4/80^+^, CD68^+^ and CD11b^+^ macrophages, the DC subsets FDC^+^ and CD11c^+^, B220^+^ B cells, and CD3^+^ T cells. Bar: 100 *μ*m.

We first tested the cytotoxicity of clodronate and clodrolip on freshly isolated murine peritoneal macrophages, human umbilical vein endothelial cells (HUVEC), murine F9 teratocarcinoma and human A637 rhabdomyosarcoma cells *in vitro*. Treatment with clodrolip killed peritoneal macrophages in a concentration-dependent manner resulting in an IC_50_ of 2.8 mM or 1 mg ml^−1^ (Figure 1A). HUVEC, F9 or A673 cells were not affected upon incubation with clodrolip at the IC_50_ (Figure 1B).

Immunohistochemical analysis of spleens of Sv129 mice (Figure 1C) showed that clodrolip treatment caused selective depletion of activated F4/80^+^ red pulp, MOMA1^+^ metallophilic marginal zone, ER-TR 9^+^ marginal zone and CD11b^+^ macrophages. CD68^+^ macrophages were not depleted, but clodrolip treatment caused clustering of CD68^+^ cells (see Supplementary Figure s1A, B). The FDC^+^ and CD11c^+^ dendritic myeloid cell subsets and the B220^+^ B- and CD3^+^ T cells were not affected. Clodronate did not cause any histologically visible depletion of the analysed cell populations in the spleen (Figure 1C and Supplementary Figure s1A, B).

### Clodrolip promotes macrophage depletion and inhibits tumour growth

**Figure 2:**
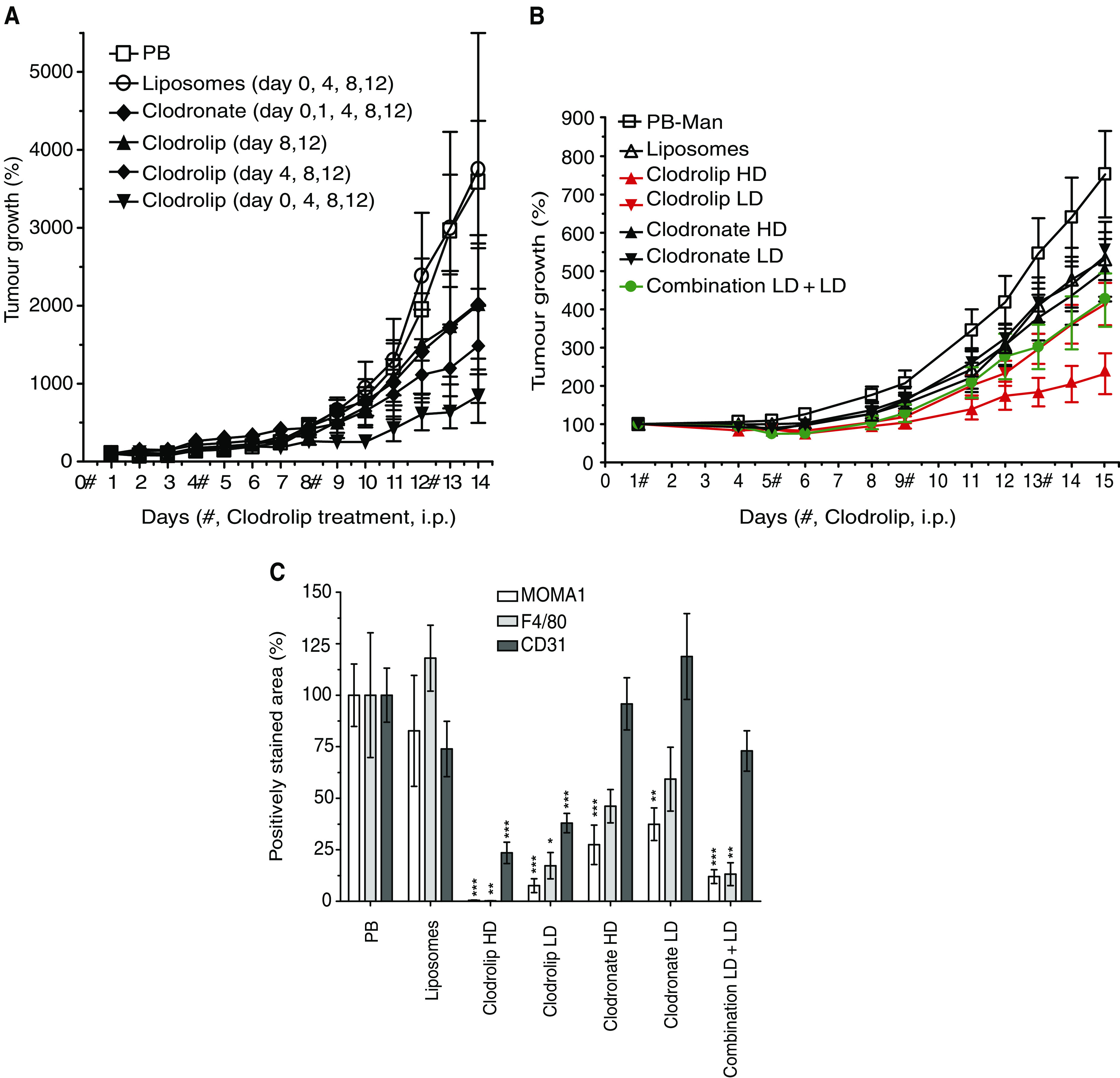
Effects of clodronate treatment on F9 teratocarcinoma growth. (**A**) Tumour-bearing mice (6–8 Sv129 mice group^−1^) were treated by i.p. injection starting 6 h after tumour cell inoculation, followed by treatments every 4 days (days 0, 4, 8 and 12) at the same dosage as in Figure 1B). In two groups, clodrolip therapy was started on days 4 or 8 after tumour cell inoculation. Values represent tumour growth±s.e.m. (*n*=6–8). Relative percentual tumour growth was normalised to day 1. (**B**) Comparison of clodronate with clodrolip treatment in the A673 rhabdomyosarcoma model. Tumour-bearing mice (6–8 CD-1 nude mice group^−1^) were treated four times every 4 days starting on day 1 (days 1, 5, 9 and 13, i.p.). Treatment doses were as in Figure 1B for the high-dose (HD) groups. The dosage for low-dose (LD) groups was 1 mg 20 g^−1^ mouse body weight as initial dose, followed by 0.5 mg for the subsequent doses. Values shown represent tumour growth±s.e.m. (*n*=6–8). Relative percentual tumour growth was normalised to day 1. (**C**) Immunohistochemical quantification of F4/80^+^, MOMA1^+^ TAMs and CD31^+^ endothelial cells (blood vessels) in A673 tumours. Three to five tumour sections were stained with the corresponding antibodies and F4/80^+^, MOMA1^+^ and CD31^+^ areas in three randomly selected fields of each section were quantified. Bars indicate the calculated averages±s.e.m. (*n*=9–15) in percent referred to the PB control sections. Statistical analysis: ^*^P<0.05; ^**^P<0.01 and ^***^P<0.001. Individual *P*-values: F4/80, clodrolip HD, ^**^P=0.0032; clodrolip LD, ^*^P=0.0138; clodronate HD and LD, NS and combination LD+LD, ^**^P=0.01. MOMA1, clodrolip HD and LD, ^***^P<0.0001; clodronate HD, ^***^P=0.0005; clodronate LD, ^**^P=0.0014 and combination LD+LD, ^***^P<0.0001. CD31, clodrolip HD and LD, ^***^P< 0.0002, clodronate HD and LD and combination LD+LD, NS and clodrolip HD or LD *vs* clodronate HD, ^***^P<0.0003.

We next studied the effects of clodrolip on tumour progression and angiogenesis in the highly vascularised and fast-growing syngeneic F9 teratocarcinoma mouse tumour model. Mice were treated i.p. with PB, empty liposomes, clodronate or clodrolip. The initial clodronate dose was 2 mg 20 g^−1^ mouse body weight, followed by 1 mg 20 g^−1^ mouse body weight given every 4 days. Therapy onset was 6 h after tumour cell inoculation. In two groups, clodrolip therapy was delayed to days 4 and 8, respectively. Clodrolip treatment inhibited tumour growth, even at the most delayed therapy onset (days 8 and 12) (Figure 2A and Supplementary Figure s2A). The most effective growth inhibition (74%, *P*=0.0185) was obtained with an early treatment on days 0, 4, 8 and 12. Clodronate (5 mg) given on days 0, 4, 8, 12 had an insignificant inhibitory effect of 45% (*P*=0.21), comparable to the days 4, 8 and 12 delayed clodrolip schedule (49%, *P*=0.167). The delayed onset of therapy at time points where tumours were already established and well vascularised (day 4 or 8) resulted in less pronounced growth inhibition, suggesting that macrophage depletion in large tumours is not sufficient to efficiently inhibit tumour growth.

To further elucidate the effect of clodronate on TAM depletion, we performed a therapy experiment in the less aggressively growing human A673 rhabdomyosarcoma xenograft model. Clodronate was applied with two dosage schemes, high dose (HD, 5 mg total dose) and low dose (LD, 2.5 mg total dose). As shown in Figure 2B (and in Supplementary Figure s2B), the most effective growth inhibition (66%, *P*=0.028) was obtained with HD clodrolip application, whereas clodronate HD therapy was less active (29%, *P*=0.146). Interestingly, clodrolip plus clodronate combination therapy (LD+LD) was equally active (42%, *P*=0.027) as clodrolip LD alone (38%, *P*=0.062), suggesting that clodronate LD was ineffective. Quantification of macrophage depletion and blood vessel density by immunohistochemistry in A673 tumours revealed a superior, but dose-dependent depletion by clodrolip compared with clodronate therapy. To assess the depletion effects, four to five tumour sections from individual mice per treatment group were analysed by determining positively stained areas (pixel counts transformed to percent) in three defined regions of interest of identical size. As shown on the bar graph in Figure 2C, MOMA-1^+^ macrophages are more susceptible to clodrolip than the quantitatively larger population of the F4/80^+^ TAMs. Clodrolip was also more efficient in reducing CD31^+^ EC counts than clodronate (clodrolip HD or LD *vs* clodronate HD; *P*<0.0003). Clodrolip treatment did not cause overt toxic side effects in the F9 and the A673 mouse tumour model and we assume that the spleen is repopulated with macrophages after the end of therapy within 1–3 weeks. Likewise, the tumour stroma may be repopulated from the periphery within the same time frame. Compared to PB-treated control mice, treatment with a total dose of 5 mg of either clodronate or clodrolip did not significantly change body weight (<±5%) during the monitored time periods (see Supplementary Figure s3A, B). This suggests that treatment with clodronate produced no general toxic side effects in the mice.

### Combining clodrolip treatment with VEGF neutralisation further enhances TAM depletion and shows improved tumour inhibition

**Figure 3:**
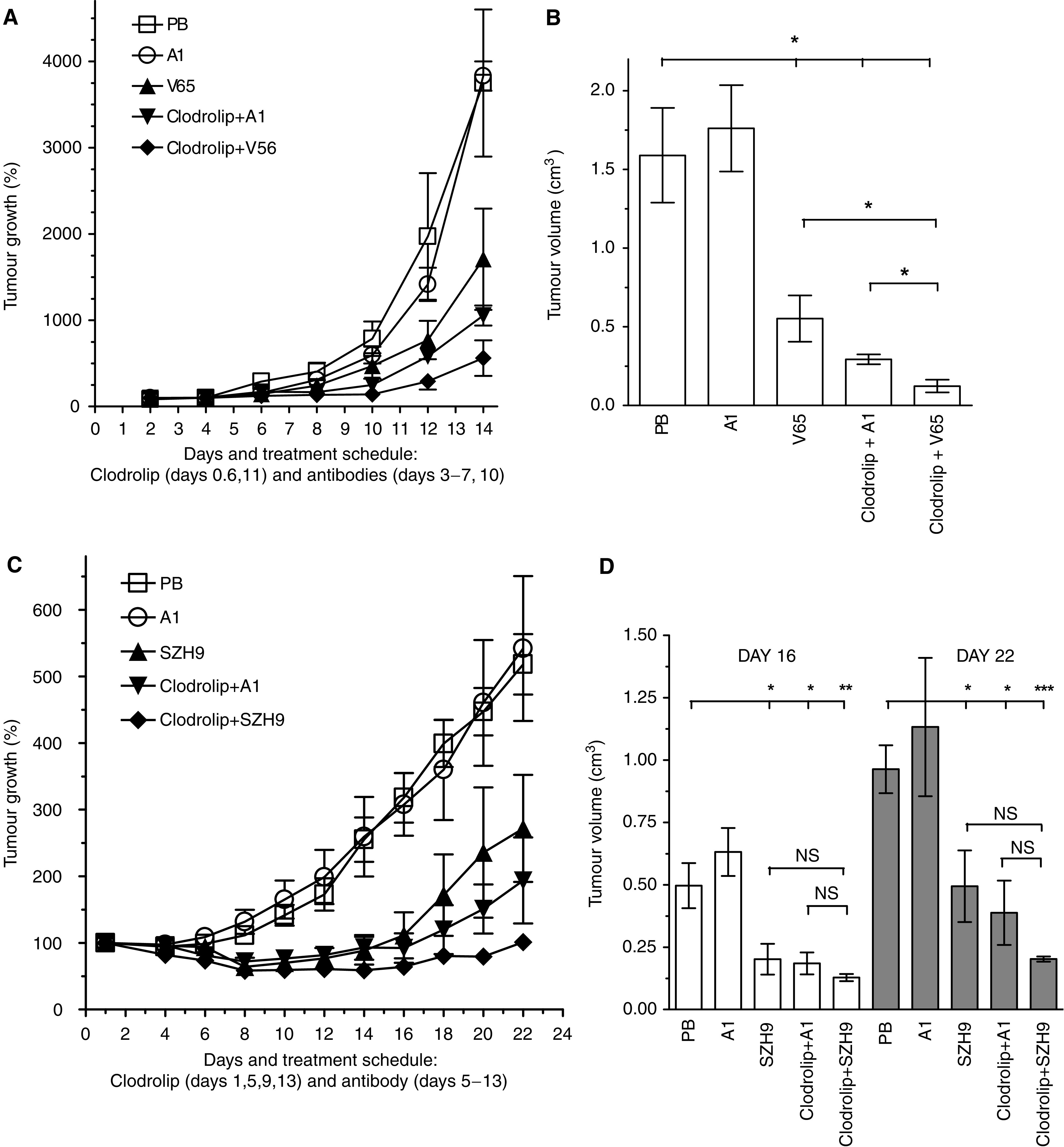
Combination of clodrolip treatment with VEGF neutralisation further enhances TAM depletion and shows improved tumour inhibition in the syngeneic F9 teratocarcinoma (**A**, **B**) and in the xenogenic human A673 rhabdomyosarcoma (**C**, **D**) models. (**A**) Tumour growth inhibition by clodrolip treatment in combination with the anti-mVEGF Ab V65. F9 tumour-bearing mice (3–5 group^−1^) were treated with PB, control Ab A1, V65, clodrolip plus A1 or clodrolip plus V65. Each clodrolip dose contained initially 2 mg 20 g^−1^ mouse body weight (day 0, i.p.), followed by 1 mg (days 6 and 11, i.p) and the Abs were given at 0.5 mg (days 3–7 and 10, i.v.). Values represent the mean of treated mice±s.e.m. (*n*=3–5). Relative percentual tumour growth was normalised to day 1. (**B**) Bar graph of tumour volumes measured on day 14, showing the calculated averages±s.e.m. (*n*=3–5). Statistical analysis: ^*^P<0.05; V65 *vs* clodrolip+V65, *P*=0.0118 and clodrolip+A1 *vs* clodrolip+V65, *P*=0.014. (**C**) Tumour growth inhibition by the combination treatment in A673 rhabdomyosarcomas. Tumour-bearing mice (6–8/group) were treated with PB, control Ab A1, SZH9 Ab (days 5–13, i.v.), clodrolip plus A1 or clodrolip plus SZH9 (days 1, 5, 9 and 13, i.p.) as in (**A**). Values represent the mean of treated mice±s.e.m. (*n*=6–8). (**D**) Bar graph of tumour growth inhibition measured at days 16 (open bars) and 22 (grey bars) showing the tumour volumes as calculated averages±s.e.m. Statistical analysis: individual *P*-values for day 16, ^*^P=0.039, ^**^P=0.015, ^***^P=0.009; for day 22, ^*^P=0.026, ^**^P=0.007, ^***^P=0.0002; NS, not significant. VEGF-neutralising properties and pharmacokinetic data of SZH9 are shown in Supplementary Figures s4 and s5.

Recognising that clodrolip-mediated depletion of TAMs might not be effective enough for complete suppression of tumour growth, we combined clodrolip with an antiangiogenic therapy by systemic application of anti-VEGF antibodies. In the F9 model, mice were either treated with PB, an unspecific control antibody A1, with the anti-mVEGF Ab V65 (Vitaliti *et al*, 2000) or with antibody combined with clodrolip. Clodrolip therapy was started 6 h after tumour cell inoculation, followed by additional applications on days 6 and 11 (Figure 3A). Clodrolip-mediated TAM depletion decreased tumour volumes (Figure 3B) significantly by 82% (*P*=0.003). Blocking VEGF with V65 antibody reduced tumour volumes by 65% (*P*=0.047). The combination protocol resulted in a significantly higher reduction of 92% (*P*=0.001), corresponding to 10 and 28% higher values compared to the single treatments, respectively.

Encouraged by the results obtained with V65, we reasoned that a VEGF-blocking antibody that neutralises both tumour cell-secreted human VEGF and mouse VEGF expressed by stromal host cells, such as macrophages, ECs and fibroblasts, would be advantageous to prevent the growth of xenograft tumours. Thus, we generated a mouse–human crossreactive anti-VEGF scFv recombinant antibody SZH9 and functionalised it at its carboxy terminus to allow further derivatisation and dimerisation (Marty *et al*, 2002).

To evaluate whether SZH9 would neutralise VEGF and consequently block angiogenesis *in vivo*, we used the chick chorioallantois membrane assay (CAM) (Scheidegger *et al*, 1999). Vascular endothelial growth factor-induced angiogenesis was reproducibly inhibited by coincubation with SZH9. Compared to the control antibody A1, vessel sprouting induced by VEGF_164_ was inhibited by SZH9 (see Supplementary Figure s4A). Additionally, pharmacokinetic properties of SZH9 were assessed in a biodistribution experiment in A673 tumour-bearing mice (see Supplementary Figure s4B). Radio-iodinated SZH9 was rapidly eliminated from blood with a biphasic profile resulting in a blood distribution half-life of *t*_1/2*α*_=0.2 h and an elimination half-life of *t*_1/2*β*_=3.3 h. A peak accumulation level of approximately 3% of the injected dose was reached in the tumour 4 h after application. Unlike IgG antibodies, SZH9 did not accumulate to significant levels in the liver or other organs. Further properties of the SZH9 antibody are summarised in the Supplementary Figure s5A–C and s5D–G.

The superior tumour inhibitory effect of combined clodrolip/antibody therapy was confirmed in the A673 tumour model (Figure 3C). The antibodies SZH9 or A1 were given in combination with clodrolip on days 5–13 every 24 h. Clodrolip treatment decreased tumour volumes by 63% (*P*=0.015) and treatment with SZH9 by 59% (*P*=0.04). The combination therapy was again clearly better than the single treatments resulting in growth inhibition by 74% (*P*=0.009; Figure 3D; left panel, day 16). In both tumour models, the combination therapy resulted in drastic tumour growth inhibition with nearly complete suppression in the strongly VEGF-dependent A673 model that persisted up to 9 days after ending treatment (as shown in Figure 3C and D; right panel, day 22).

### Immunohistochemical analysis of TAM depletion

**Figure 4:**
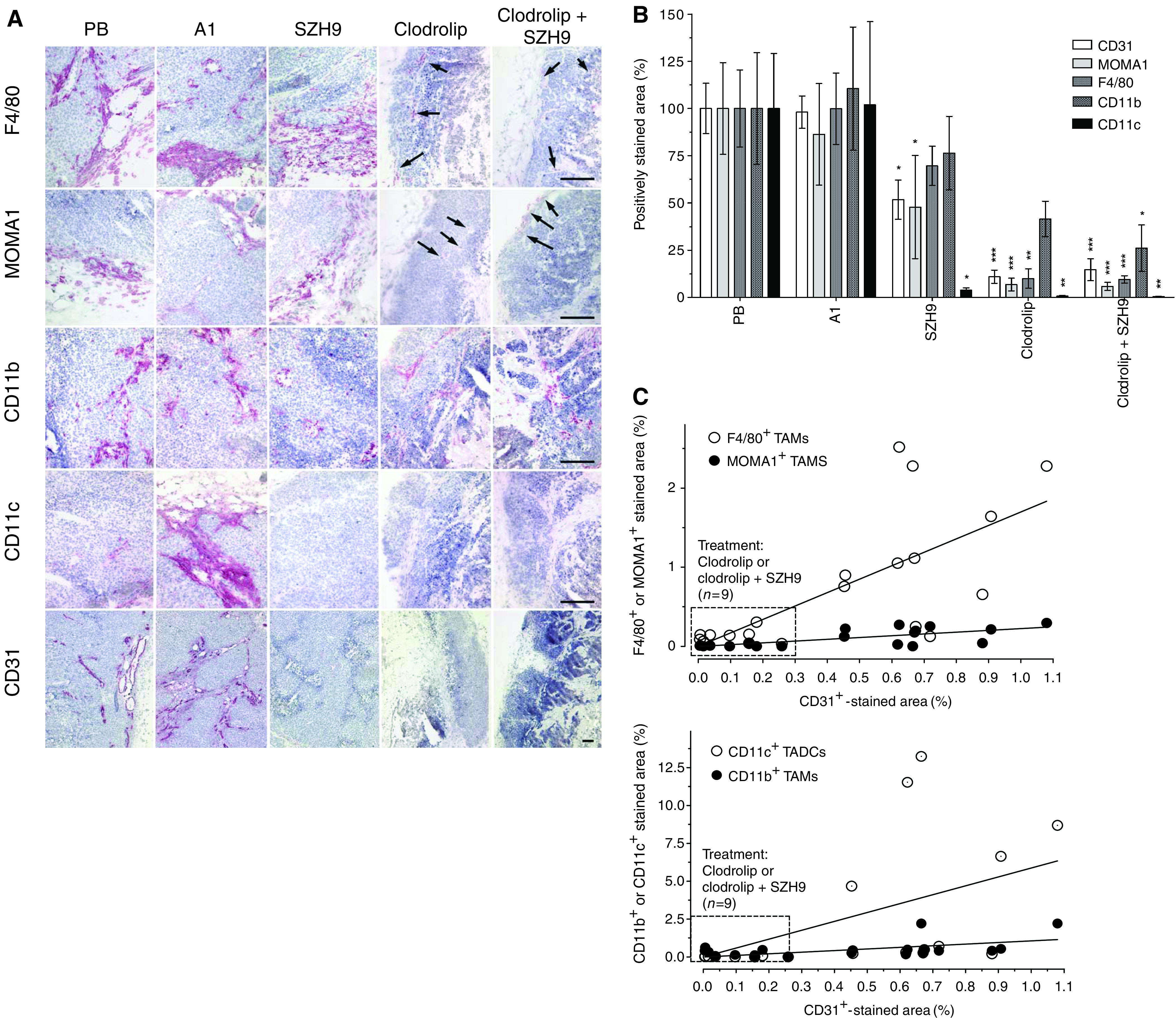
Immunohistochemical analysis of TAM depletion and correlation to blood vessel density. (**A**) A673 tumour sections obtained from mice injected with PB, A1, SZH9, clodrolip alone or in combination with SZH9 on days 16 after onset of treatment were analysed by IHC. Depletion of activated F4/80^+^, marginal zone MOMA1^+^ and CD11b^+^ TAMs and of CD11c^+^ tumour-associated dendritic cells (TADCs) is shown. Blood vessel staining is shown by staining of CD31^+^ endothelial cells. Arrows show cells that were not depleted (or repopulated) after the end of therapy. Bars: 100 *μ*m. (**B**) Quantification of F4/80^+^, MOMA1^+^, CD11b^+^ TAMs, CD11c^+^ TADCs and CD31^+^ ECs (blood vessels) at day 22 (see Figure 3C and D). Three to five tumour sections were stained and quantified as indicated in Figure 2C. The bars show the calculated averages±s.e.m. (*n*=9–15) in percents referred to the PB control sections. Statistical analysis: ^*^P<0.05; ^**^P<0.01 and ^***^P<0.001. Individual *P*-values: F4/80, SZH9, NS; clodrolip, ^**^P=0.0003; clodrolip+SZH9, ^***^P<0.0001. MOMA1, SZH9, ^*^P=0.043; clodrolip, ^***^P=0.0001; clodrolip+SZH9, ^***^P<0.0001. CD31, SZH9, ^*^P=0.03; clodrolip, ^***^P<0.0001; clodrolip+SZH9, ^***^P<0.0001. CD11b, SZH9, NS; clodrolip, *P*=0.033; clodrolip+SZH9, ^*^P=0.03. CD11c, SZH9, ^*^P=0.019; clodrolip, ^**^P=0.0055 and clodrolip+SZH9, ^**^P=0.0002. (**C**) Correlation of TAM depletion (F4/80^+^, MOMA-1^+^, CD11b^+^) and of CD11c^+^ TADCs *vs* vessel density (CD31^+^ cells). The dots represent values of positively stained areas from individual tumours, showing the clear separation of clodrolip treated (box) compared to other groups (PB, Ab A1, Ab SZH9). Statistical analysis (Pearson correlation, *n*=20): F4/80, *r*=0.701, *P*=0.0006; MOMA1, *r*=0.711, *P*=0.0004; CD11b, *r*=0.50, *P*=0.025; CD11c, *r*=0.531, *P*=0.016.

Immunohistochemical analysis of A673 tumour sections from mice killed 3 and 9 days, respectively, after ending treatment was performed to evaluate macrophage inflitration and vessel density in the tumour mass (days 16 and 22, see Figure 3C and D). Substantial depletion of tumour-associated F4/80^+^ and MOMA1^+^ macrophages was observed 3 days after the last clodrolip application (Figure 4A) that persisted up to the end of the experiment (Figure 4B). Immunohistochemical analysis of tumour sections for CD11b^+^ TAMs and CD11c^+^ TADCs gave unexpected results. Whereas both cell populations remained unaffected in the spleen (see Figure 1B and Supplementary Figure s1), treatment of A673 tumours with SZH9, clodrolip or with a combination of both reagents strongly reduced the CD11b^+^ cell population, whereas CD11c^+^ TADCs were completely eradicated (Figure 4A and B).

To assess the effect of these treatments on tumour vascularisation, we analysed vessel density by CD31^+^ staining. At 3 days after the end of therapy (day 16), tumour blood vessels were virtually undetectable and significant reduction in vessel density was observed even 9 days later (Figure 4A and B).

Quantification and statistical analysis of the IHC results from A673 tumour sections of individual mice showed depletion of tumour-associated F4/80^+^ by 93% (*P*=0.0003) and of MOMA1^+^ macrophages by 90% (*P*=0.0001) in clodrolip-treated animals. Interestingly, even single treatment with SZH9 resulted in depletion of F4/80^+^ by 52% (*P*=0.18) and of MOMA1^+^ macrophages by 30% (*P*=0.043, Figure 4B). We explain this effect by the inhibition of VEGF acting as an attractant for macrophages. The combination therapy was not significantly superior over the single treatments with respect to F4/80^+^ and MOMA1^+^ macrophage depletion, presumably owing to the almost quantitative depletion obtained with clodrolip alone (Figure 4A and B). CD11b^+^ TAMs were reduced by 24% (*P*=0.114) after SZH9, by 59% (*P*=0.033) after clodrolip and by 74% (*P*=0.029) after clodrolip plus SZH9 treatment, respectively (Figure 4A and B). Finally, significant depletion of CD11c^+^ TADCs by 96% (*P*=0.043) after SZH9, by 99% (*P*=0.0034) after clodrolip and by 99% (*P*=0.0006) after clodrolip plus SZH9 treatment, respectively, was observed (Figure 4A and B).

Quantification of CD31^+^ ECs on day 22 showed that SZH9 monotherapy reduced vessel density by 48% (*P*=0.03), clodrolip alone by 89% (*P*<0.0001), whereas the combination therapy gave an 85% (*P*<0.0001) reduction which was statistically not significantly different from clodrolip monotherapy (Figure 4B). The correlation of F4/80^+^ and MOMA1^+^ macrophage density *vs* microvessel counts (CD31^+^ cells) showed a clear separation of tumours treated with clodrolip or clodrolip plus SZH9 compared to tumours treated with SZH9 alone or with A1 or PB. (Figure 4C; top). Correlation of CD11b^+^ and CD11c^+^ cell depletion with vessel density (CD31^+^ cells) confirms these results (Figure 4C; bottom panel). CD11c^+^ TADCs, which are partially also CD11b^+^, can differentiate into endothelial-like cells in a VEGF-dependent fashion as shown before (Coukos *et al*, 2005). Thus, blocking VEGF and, at the same time, depleting TAMs significantly reduced TADCs and this presumably resulted in the drastic reduction of vessel density observed in tumour sections. A summary of the quantification of these data in A673 tumours with all IHC markers used is given in Table 1.

## DISCUSSION

Tumour-associated macrophages are derived from circulating monocytes and are activated macrophages of the polarised type II (type II or M2 macrophages) induced by IL-4, IL-13 (M2a) or IL-10 (M2c) and glucocorticoid hormones. Differential cytokine and chemokine production and coordinated temporal and spatial activities of these cells in the tumour stroma are key features of polarised macrophages and promote tumour angiogenesis and growth (Mantovani *et al*, 2004). Owing to their tumour stimulatory role, TAMs have been proposed as potential therapeutic targets (Joyce, 2005). However, direct elimination of these macrophages has not yet been exploited in a drug-based therapeutic approach. In this study, we evaluated the technique of macrophage depletion using clodronate encapsulated in small unilamellar liposomes (clodrolip) as potential therapeutic regimen. We show that this approach, combined with scFv antibody-mediated neutralisation of VEGF, leads to virtually complete elimination of F4/80^+^ and MOMA1^+^ TAMs and CD11c^+^ TADCs. TAM depletion was accompanied by significant inhibition of tumour growth in both syn- and xenogenic tumour models. From these data, we surmise that TAM depletion is indeed the cause of tumour growth inhibition. Depleting these stromal cell subsets presumably disrupts the cytokine network owing to an imbalance in the cell populations that are the source of tumour stimulatory chemokines and cytokines.

In our experiments, antiangiogenic combination therapy was successful, yet it was not able to fully suppress tumour growth, resulting in slow regrowth of tumours after the end of therapy. Depleting TAM and CD11c^+^ TADC subsets apparently did not exhaust all stromal sources of proangiogenic and tumourigenic mediators, such as for example mast cells, neutrophils, fibroblasts and other DC subsets, and possibly also pericytes. Thus, TAM depletion, as most antiangiogenic therapies, ought to be regarded as an adjuvant treatment in combination with conventional chemo- or radiotherapy. Low-dose clodrolip therapy applied at higher frequency, also termed ‘metronomic’ therapy (Kerbel and Kamen, 2004), might be superior to conventional maximal tolerated dose regimens and further improve TAM and TADC depletion. In addition, such low-dose regimens, while still keeping the levels of TAMs low, might reduce systemic immunosuppressive effects. Additional suppression of systemic side effects might be achieved by specific targeting of clodronate-liposomes to TAMs, using for example mannosylated liposomes targeted to M2 polarised macrophages that express high levels of the mannose receptor. Alternatively, clodronate-containing liposomes could be conjugated with anti-ED-B fibronectin antibodies for selective deposition of TAM-depleting liposomes in the tumour matrix (Marty *et al*, 2002).

The important role of VEGF was further supported by the distinct antiangiogenic effect obtained with the anti-VEGF antibody monotherapy, which resulted in a reduction of F4/80^+^, MOMA^+^ and CD11b^+^ TAMs and the eradication of CD11c^+^ TADCs. This implies that, due to a decrease in the number of blood vessels, macrophage and DC infiltration into the stroma is reduced, or that blocking VEGF prevents monocyte attraction, or, most likely, that both mechanisms simulatenously influence TAM accumulation in tumours. High VEGF, IL-6, IL-10, TGF*β* and M-CSF levels in the tumour microenvironment block dendritic cell differentiation and maturation. Whereas functionally mature myeloid dendritic cells induce potent tumour-associated antigen-specific immunity *in vivo*, immature myeloid cells (IMCs) either suppress regulatory T cells (T_reg_) or lead to T-cell unresponsiveness (Zou, 2005). Immature myeloid cells function as regulatory cells and are therefore an important component of the immunosuppressive networks in the tumour microenvironment. Recent findings demonstrate that IMCs in the spleen of tumour-bearing mice differentiate towards F4/80^+^ TAMs. These TAMs are able to inhibit a T-cell-mediated immune response controlled by STAT1 (Kusmartsev and Gabrilovich, 2005). Moreover, the *in vivo* ablation of CD11c^+^ dendritic cells in diphtheria-toxin transgenic mice abrogates priming of cytotoxic T-lymphocyte precursors in immune responses to cell-associated antigens, a phenomenon called cross-priming (Jung *et al*, 2002). The complexity of DC populations and their different functions in the tumour microenvironment is accentuated by the recent discovery of a population of CD11c^+^ DCs, termed vascular leucocytes. These DCs simultaneously express both endothelial and dendritic cell markers, and, depending on the microenvironment, assemble into functional blood vessels or act as antigen-presenting cells. Dendritic cell precursor-mediated vasculogenesis has also been found to be regulated through the cooperation of *β*-defensins and VEGF-A, where VEGF-A primarily induces their endothelial-like specialisation and migration to blood vessels (Coukos *et al*, 2005). Thus, removal of an important source of VEGF and concomitant blocking of VEGF with neutralising antibodies, as implemented in our therapeutic regimen, might be the major cause for the disappearance of this DC subpopulation. Chemokines and chemokine receptors have been used as targets for the development of therapeutic strategies to control inflammatory disorders, and recent results suggest that chemokine inhibitors also affect tumour growth by reducing macrophage infiltration (Balkwill, 2004). Colony stimulating factor 1 (CSF-1), a cytokine commonly produced by tumours, triggers monocyte migration (Pixley and Stanley, 2004) and blocking CSF-1 or its receptor has been shown to suppress macrophage infiltration and to reduce tumour growth (Aharinejad *et al*, 2004).

Recently, it was shown by Allavena *et al* (2005) that Yondelis (Trabectedin), a new anticancer agent of marine origin, markedly reduced the levels of proinflammatory cytokines CCL2 and IL-6 in monocytes and macrophages, thus inhibiting macrophage viability, differentiation and cytokine production.

Finally, VEGF-C production by TAMs was proposed to play a role in lymphangiogenesis and lymphatic metastasis in several human cancers (Pepper *et al*, 2003).

Taken together, our findings provide solid evidence for the importance of TAMs, and possibly also of TADCs, in the establishment of a microenvironment favouring tumour growth and dissemination. Clodronate- or other bisphosphonate liposome-mediated macrophage depletion regimens open new possibilities to study the role of tumour infiltrating cells, for example by gene expression profiling of TAM-depleted tumours. In addition, TAM depletion combined with new antiangiogenic or cytotoxic therapies is a promising new approach with high clinical potential.

## Figures and Tables

**Table 1:** Summary of IHC analysis

	**Treatment and histological scoring of A673 tumors**				
**Marker**	**PBS**	**A1**	**SZH9**	**Clodrolip**	**Clodrolip+SZH9**
F4/80	+++	+++	++	−	−
MOMA1	+++	+++	++	−	−
ER-TR 9	−	−	−	−	−
CD68^a^	+++	+++	+++	+++	+++
CD11b	+++	+++	++	++	++
CD11c	+++	+++	−	−	−
CD31	+++	+++	++	+	+
LYVE-1	−	−	−	−	−

a For IHC sections, see Supplementary Figure s4.

Scoring of TAM (F4/80, MOMA1, ER-TR 9, CD68, CD11b), TADC (CD11c) and blood-endothelial (CD31) or lymph-endothelial (LYVE-1) cell depletion efficiency. Depletion efficiencies in A673 tumours with all cell markers from IHC tumour specimens are listed (day 22, experiment shown in Figure 2B, C). Scoring of staining: +++, very strong; ++ moderate; +weak or inconsistent, −absent. Histological sections of HE- and CD68-stained A673 tumours are shown in Supplementary Figure s6A and B.

## References

[bib1] Aharinejad S, Paulus P, Sioud M, Hofmann M, Zins K, Schäfer R, Stanley RE, Abraham D (2004) Colony-stimulating factor-1 blockade by antisense oligonucleotides and small interfering RNAs suppresses growth of human mammary tumor xenografts in mice. Cancer Res 64: 5378–5384 15289345 10.1158/0008-5472.CAN-04-0961

[bib2] Allavena P, Signorelli M, Chieppa M, Erba E, Bianchi G, Marchesi F, Olimpio CO, Bonardi C, Garbi A, Lissoni A, de Braud F, Jimeno J, D'Incalci M (2005) Anti-inflammatory properties of the novel antitumor agent Yondelis (Trabectedin): inhibition of macrophage differentiation and cytokine production. Cancer Res 65: 2964–2971 15805300 10.1158/0008-5472.CAN-04-4037

[bib3] Balkwill F (2004) Cancer and the chemokine network. Nat Rev Cancer 4: 540–550 15229479 10.1038/nrc1388

[bib4] Chen JJW, Lin Y-C, Yao P-L, Yuan A, Chen H-Y, Shun C-T, Tsai M-F, Chen C-H, Yang P-C (2005) Tumor associated macrophages: the double-edged sword in cancer progression. J Clin Oncol 23: 953–964 15598976 10.1200/JCO.2005.12.172

[bib5] Coukos G, Benencia F, Buckanovich RJ, Conejo-Garcia JR (2005) The role of dendritic cell precursors in tumor vasculogenesis. Br J Cancer 92: 1182–1187 15785750 10.1038/sj.bjc.6602476 PMC2361965

[bib6] Folkman J (2003) Fundamental concepts of the angiogenic process. Curr Mol Med 3: 643–651 14601638 10.2174/1566524033479465

[bib7] Friguet B, Chaffotte AF, Djavadi-Ohaniance L, Goldberg ME (1985) Measurements of the true affinity constant in solution of antigen-antibody complexes by enzyme-linked immunosorbent assay. J Immunol Meth 77: 305–319 10.1016/0022-1759(85)90044-4 3981007

[bib8] Giraudo E, Inoue M, Hanahan D (2004) An amino-bisphosphonate targets MMP-9-expressing macrophages and angiogenesis to impair cervical carcinogenesis. J Clin Invest 114: 623–633 15343380 10.1172/JCI22087 PMC514591

[bib9] Joyce JA (2005) Therapeutic targeting of the tumor microenvironment. Cancer Cell 7: 513–520 15950901 10.1016/j.ccr.2005.05.024

[bib10] Jung S, Unutmaz D, Wong P, Sano GJ, De los Santos K, Sparwasser T, Wu S, Vuthoori S, Ko K, Zavala F, Pamer EG, Littman DR, Lang RA (2002) *In vivo* depletion of CD11c^+^ dendritic cells abrogates priming of CD8^+^ T cells by exogenous cell-associated antigens. Immunity 17: 211–220 12196292 10.1016/s1074-7613(02)00365-5 PMC3689299

[bib11] Kerbel RS, Kamen BA (2004) The anti-angiogenic basis of metronomic chemotherapy. Nat Rev Cancer 4: 423–436 15170445 10.1038/nrc1369

[bib12] Khong HT, Restifo NP (2002) Natural selection of tumor variants in the generation of ‘tumor escape’ phenotypes. Nat Immunol 3: 999–1005 12407407 10.1038/ni1102-999 PMC1508168

[bib13] Kusmartsev S, Gabrilovich DI (2005) STAT1 signaling regulates tumor-associated macrophage-mediated T cell deletion. J Immunol 174: 4880–4891 15814715 10.4049/jimmunol.174.8.4880

[bib14] Mantovani A, Allavena P, Sica A (2004) Tumour-associated macrophages as a prototypic type II polarised phagocytic population: role in tumour progression. Eur J Cancer 40: 1660–1667 15251154 10.1016/j.ejca.2004.03.016

[bib15] Marty C, Meylan C, Schott H, Ballmer-Hofer K, Schwendener RA (2004) Enhanced heparin sulphate proteoglycan-mediated uptake of cell-penetrating peptide-modified liposomes. Cell Mol Life Sci 61: 1785–1794 15241554 10.1007/s00018-004-4166-0 PMC11146021

[bib16] Marty C, Odermatt B, Schott H, Neri D, Ballmer-Hofer K, Klemenz R, Schwendener RA (2002) Cytotoxic targeting of F9 teratocarcinoma tumours with anti-ED-B fibronectin scFv antibody modified liposomes. Br J Cancer 87: 106–112 12085265 10.1038/sj.bjc.6600423 PMC2364274

[bib17] Maruyama K, Masaaki I, Cursiefen C, Jackson DG, Keino H, Tomita M, van Roojien N, Takenaka H, D'Amore PA, Stein-Streilein J, Losordo DW, Streilein JW (2005) Inflammation-induced lymphangiogenesis in the cornea arises from CD11b-positive macrophages. J Clin Invest 115: 2363–2372 16138190 10.1172/JCI23874 PMC1193872

[bib18] Mrkic B, Odermatt B, Klein MA, Billeter MA, Pavlovic J, Cattaneo R (2000) Lymphatic dissemination and comparative pathology of recombinant measles viruses in genetically modified mice. J Virol 74: 1364–1372 10627547 10.1128/jvi.74.3.1364-1372.2000 PMC111471

[bib19] Murdoch C, Giannoudis A, Lewis CE (2004) Mechanisms regulating the recruitment of macrophages into hypoxic areas of tumors and other ischemic tissues. Blood 104: 2224–2234 15231578 10.1182/blood-2004-03-1109

[bib20] Ochsenbein AF (2005) Immunological ignorance of solid tumours. Springer Semin Immunol 27: 19–35 10.1007/s00281-004-0192-0 15965711

[bib21] Pepper MS, Tille J-C, Nisato R, Skobe M (2003) Lymphangiogenesis and tumor metastasis. Cell Tissue Res 314: 167–177 12883995 10.1007/s00441-003-0748-7

[bib22] Pini A, Viti F, Santucci A, Carnemolla B, Zardi L, Neri P, Neri D (1998) Design and use of a phage display library. Human antibodies with subnanomolar affinity against a marker of angiogenesis eluted from a two-dimensional gel. J Biol Chem 273: 21769–21776 9705314 10.1074/jbc.273.34.21769

[bib23] Pixley FJ, Stanley ER (2004) CSF-1 regulation of the wandering macrophage: complexity in action. Trends Cell Biol 14: 628–638 15519852 10.1016/j.tcb.2004.09.016

[bib24] Podar K, Anderson KC (2005) The pathophysiologic role of VEGF in hematologic malignancies: therapeutic implications. Blood 105: 1383–1395 15471951 10.1182/blood-2004-07-2909

[bib25] Pollard JW (2004) Tumour-educated macrophages promote tumour progression and metastasis. Nat Rev Cancer 4: 71–78 14708027 10.1038/nrc1256

[bib26] Rogers MJ, Gordon S, Benford HL, Coxon FP, Luckman SP, Monkkonen J, Frith JC (2000) Cellular and molecular mechanisms of action of bisphosphonates. Cancer 88: 2961–2978 10898340 10.1002/1097-0142(20000615)88:12+<2961::aid-cncr12>3.3.co;2-c

[bib27] Ross JR, Saunders Y, Edmonds PM, Patel S, Wonderling D, Normand C, Broadley K (2004) A systematic review of the role of bisphosphonates in metastatic disease. Health Technol Assess 8: 1–176 10.3310/hta8040 14960258

[bib28] Scheidegger P, Weigelhofer W, Suarez S, Kaser-Hotz B, Steiner R, Ballmer-Hofer K, Jaussi R (1999) Vascular endothelial growth factor (VEGF) and its receptors in tumor-bearing dogs. Biol Chem 380: 1449–1454 10661874 10.1515/BC.1999.187

[bib29] Seiler P, Aichele P, Odermatt B, Hengartner H, Zinkernagel RM, Schwendener RA (1997) Crucial role of marginal zone macrophages and marginal zone metallophils in the clearance of lymphocytic choriomeningitis virus infection. Eur J Immunol 27: 2626–2633 9368619 10.1002/eji.1830271023

[bib30] Tyner JW, Uchida O, Kajiwara N, Kim EY, Patel AC, O'Sullivan MP, Walter MJ, Schwendener RA, Cook DN, Danoff TM, Holtzman MJ (2005) CCL5/CCR5 interaction provides anti-apoptotic signals for macrophage survival during viral infection. Nat Med 11: 1180–1187 16208318 10.1038/nm1303 PMC6322907

[bib31] Vitaliti A, Wittmer M, Steiner R, Wyder L, Neri D, Klemenz R (2000) Inhibition of tumor angiogenesis by a single-chain antibody directed against vascular endothelial growth factor. Cancer Res 60: 4311–4314 10969766

[bib32] Vosseler S, Mirancea N, Bohlen P, Mueller MM, Fusenig NE (2005) Angiogenesis inhibition by vascular endothelial growth factor receptor-2 blockade reduces stromal matrix metalloproteinase expression, normalizes stromal tissue, and reverts epithelial tumor phenotype in surface heterotransplants. Cancer Res 65: 1294–1305 15735015 10.1158/0008-5472.CAN-03-3986

[bib33] Workman P, Twentyman PR, Balkwill F, Balmain A, Chaplin DJ, Double JA, Embleton MJ, Newell D, Raymond R, Stables J, Stephens T, Wallace J (1998) United Kingdom Co-ordinating Committee on Cancer Research (UKCCCR) Guidelines for the Welfare of Animals in Experimental Neoplasia (Second Edition). Br J Cancer 77: 1–10 10.1038/bjc.1998.1 PMC2151254 9459138

[bib34] Zou W (2005) Immunosuppressive networks in the tumor environment and their therapeutic relevance. Nat Rev Cancer 5: 263–274 15776005 10.1038/nrc1586

