# Supplementary material for: Clodronate-liposome-mediated depletion of tumour-associated macrophages: a new and highly effective antiangiogenic therapy approach
Source: Br J Cancer. 2006 Jul 11;95(3):272–81. doi: 10.1038/sj.bjc.6603240 (PMC2360657; doi:10.1038/sj.bjc.6603240)
Supplement: Supplementary Figures [file 95-6603240x1.pdf]

Supplementary data, Zeisberger *et al.*

Figure 1, A,B

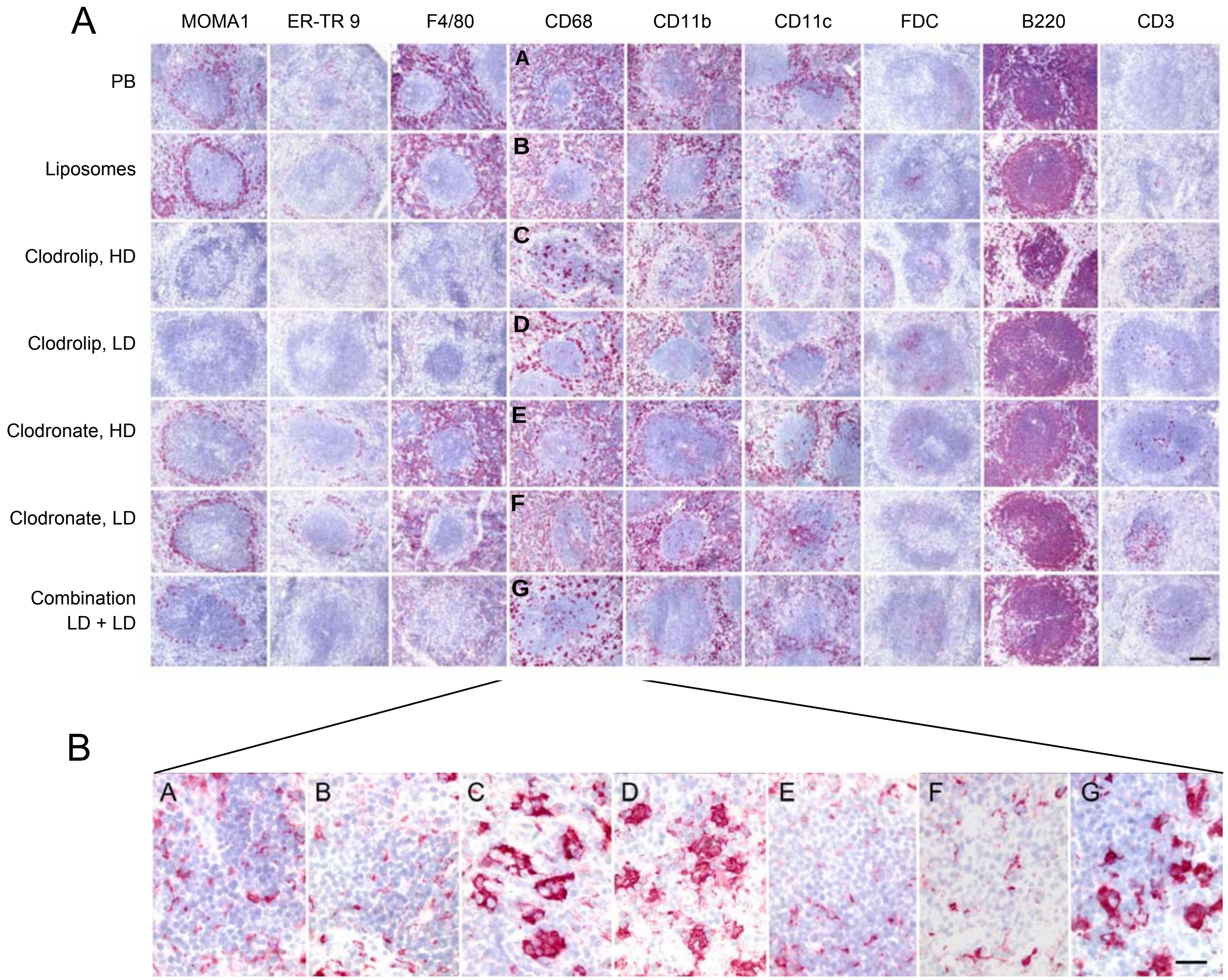

Effect of clodronate and clodrolip therapy in spleens of A673 rhabdomyosarcoma bearing mice.

A, Staining pattern of the macrophage markers MOMA-1, ER-TR 9, F4/80, CD11b and CD68, the dendritic cell markers CD11c and FDC and the B- and T-cell markers are shown for the different treatments (see Fig 2, C). Bar, 100 μm. HD, high dose; LD, low dose. B, Magnification of CD68 stained sections. Panels C,D and G show clodrolip treated spleens where clustering of CD68 positive macrophages was observed. PB, empty liposomes and clodronate did not cause this effect. Bar, 250 μm.

Fig. s2, A

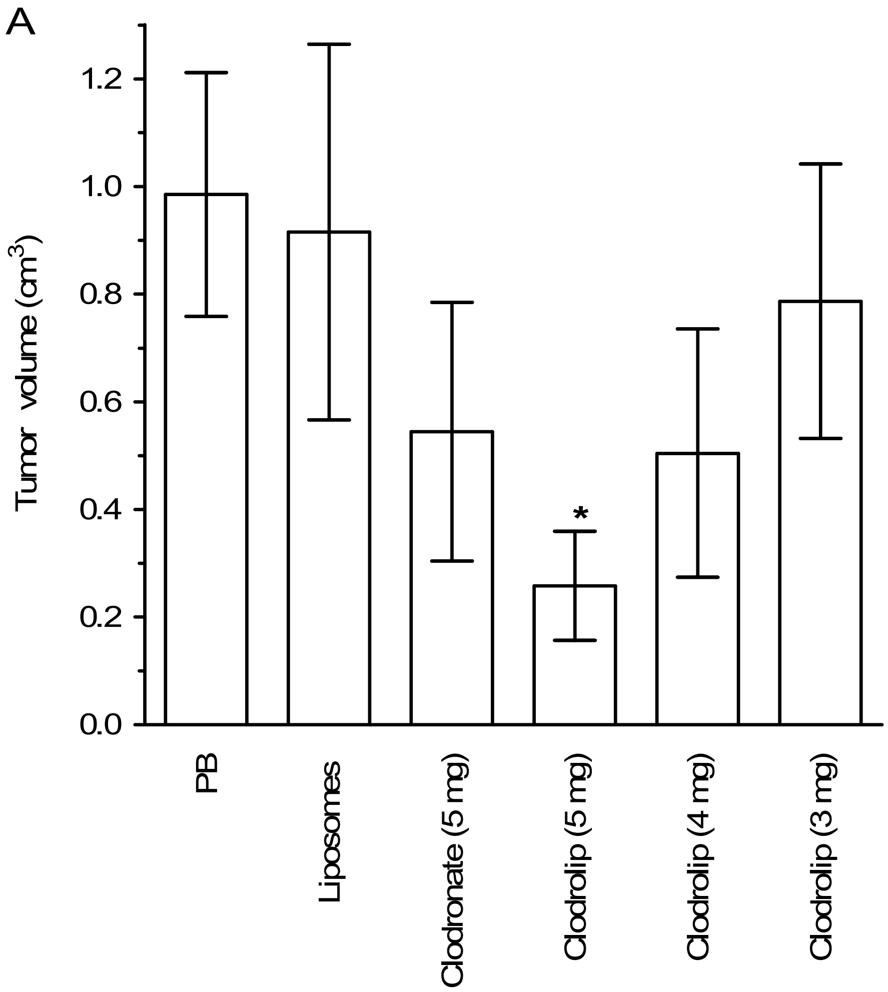

Fig s2 A, **Effects of clodronate and clodrolip treatment on F9 teratocarcinoma growth**. Bar graph of tumor volumes at day 14 ± SEM (n = 6 - 8). Statistical analysis: $P < 0.05$.

Fig. s2, B

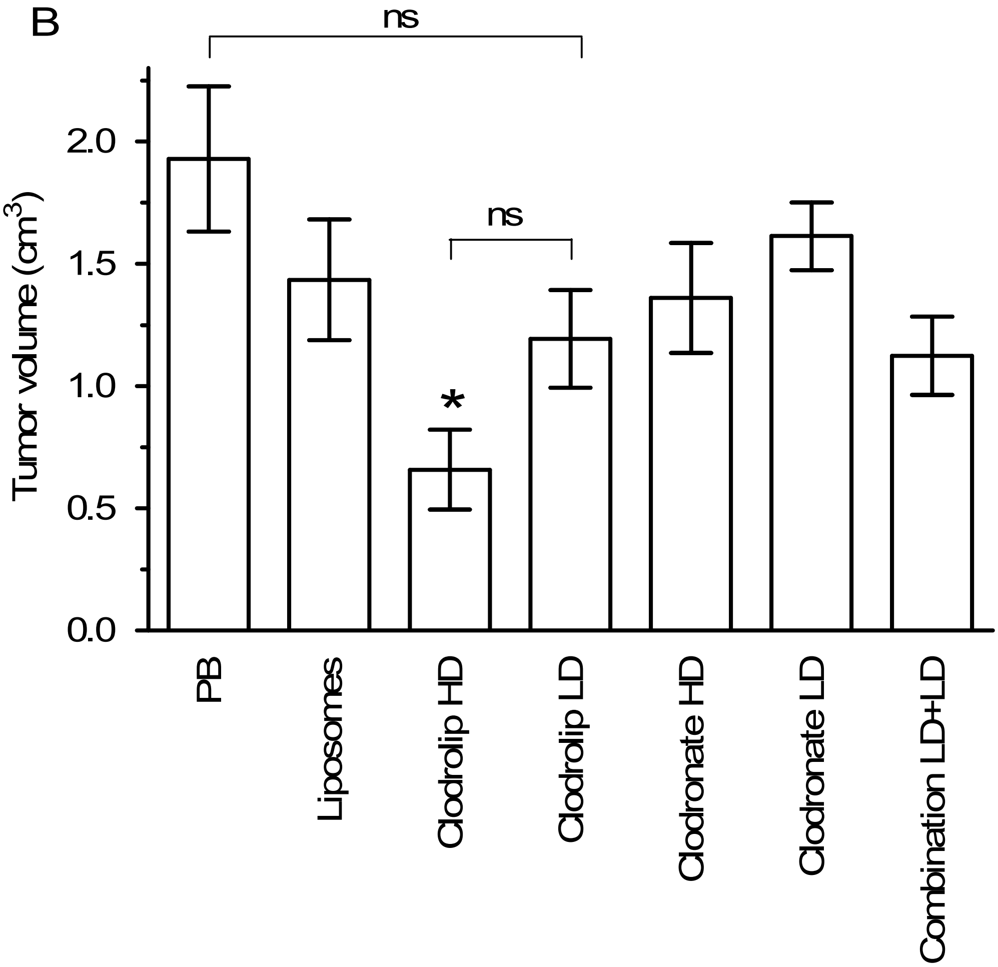

Fig. s2 B, **Effects of clodronate and clodrolip treatment on F9 teratocarcinoma growth** Dosage: HD, 2 + 1 + 1 + 1 mg/ml; LD, 1 + 0.5 + 0.5 + 0.5 + 0.5 mg/ml. Bar graph of tumor volumes at day 15 ± SEM (n = 6 - 8). Statistical analysis;. P = 0.005; ns, not significant.

Fig. s3 A, B

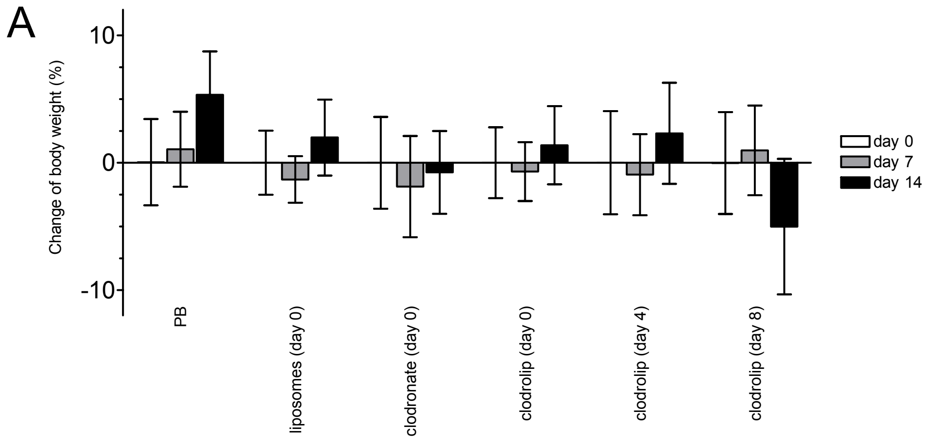

Fig. s3 A, **Body weight changes recorded during the F9 teratocarcinoma treatment.** Data from experiment shown in Fig. 2 A are shown. Values represent the mean of 6 - 8 treated mice, ± SEM. Calculated *P* values (treated *vs*. PB) resulted in insignificant changes.

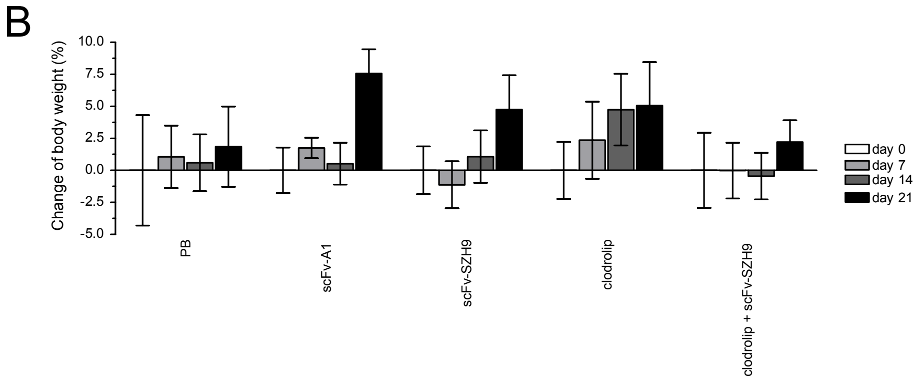

Fig. s3 B, **Body weight changes recorded during the A673 rhabdomyosarcoma treatment experiment.** Data from experiment shown in Fig. 2 B. are shown. Values represent the mean of 6 - 8 treated mice, ± SEM. Calculated *P* values (treated *vs*. PB) resulted in insignificant changes.

Fig. s4, A

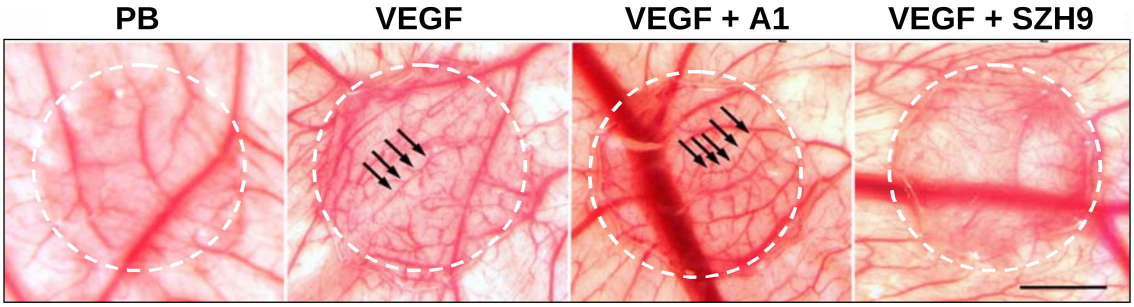

Fig. s4 A, **Chicken CAM angiogenesis assay.** CAMs of chick embryos (day 10) were incubated for 72 h with PB, cfVEGF164 and cfVEGF164 (3 mg) co-incubated either with the A1 or the SZH9 (50 mg) Abs. Angiogenesis was observed by sprouting of new blood vessels from larger pre-existing vessels (indicated by arrows). Angiogenic effects were not visible when VEGF was blocked with SZH9 Ab. Dashed circles outline the methylcellulose disc onto which the compounds were applied. Bar: 2.5 mm. b) Pharmacokinetic profile of 125I-labelled SZH9 Ab in A673 tumor bearing mice. Tumor, liver, kidney and blood levels are shown as percent injected dose per gram (%ID/g).

Chick chorioallantoic membrane (CAM) assay: Experiments were performed on chicken embryos grown by the *ex ovo* culture method (Biol. Chem. 1999;380:1449-54). Fertilized chicken eggs (Lohman LSL strain) were from Animalco (Staufen, Switzerland) and kept in a humidified incubator at 37.9°C. Methylcellulose discs (0.5%, w/v; 5 mm Ø) supplied with 3 μg cfVEGF164 in presence of 50 μg scFv Ab were grafted onto the growing CAM at day 10 of embryonic development. Parallel experiments were performed with plain discs, discs supplied with PB or 3 μg cfVEGF164. To enhance contrast, the CAM was underlayed with a lipid emulsion (Lipovenös 20%, Fresenius) and the angiogenic response evaluated by three independent observers under a stereo microscope.

Fig. s4, B

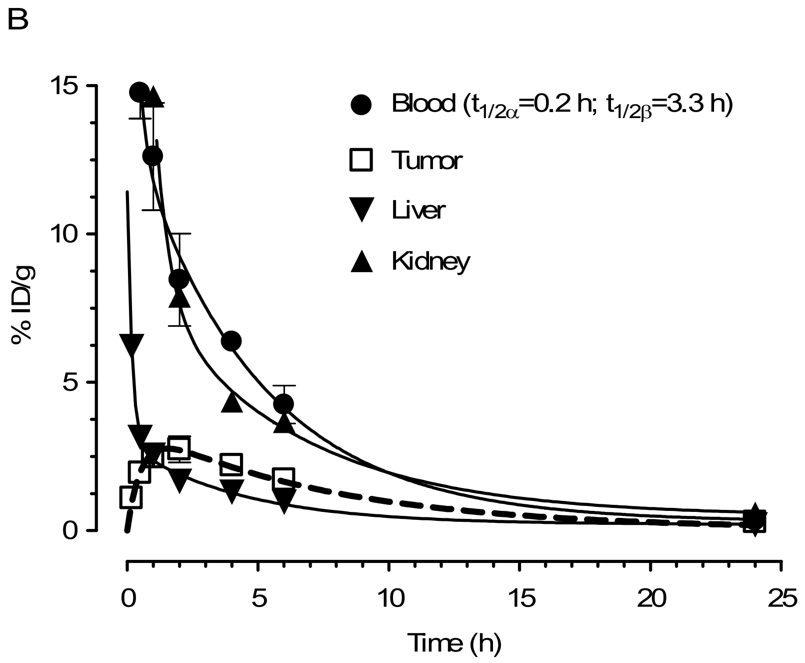

Fig. s4, B, **Pharmacokinetic profile of ^{125}I-labelled SZH9 Ab (3 μg in 0.1 ml; i.v.) in A673 tumor bearing mice.** Tumor, liver, kidney and blood levels are shown as percent injected dose per gram (%ID/g).

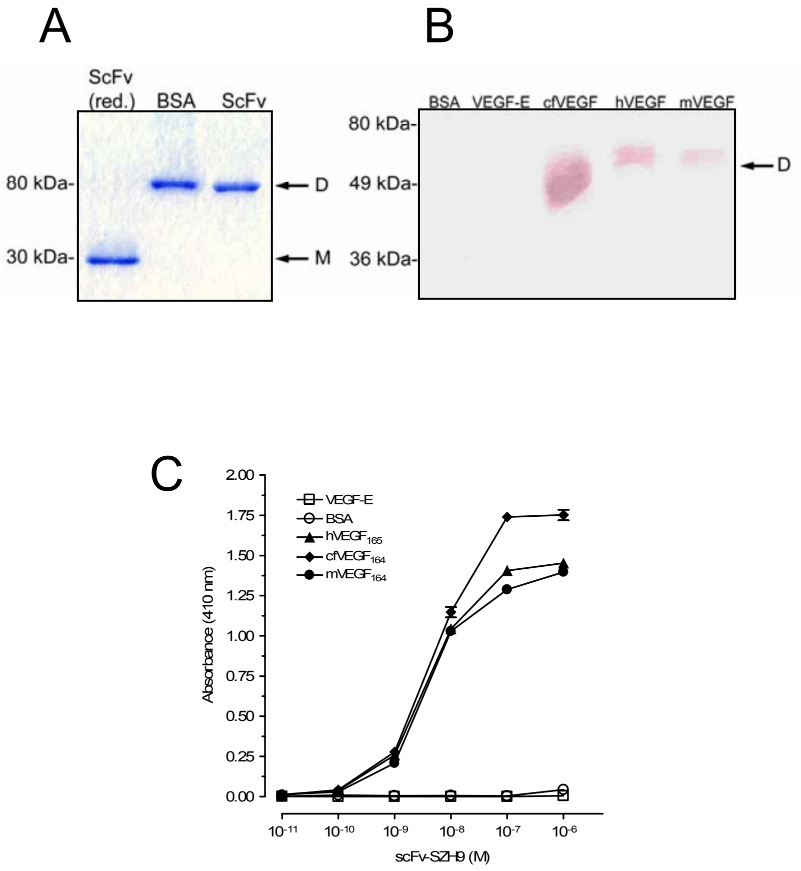

Fig. s5 A-C, **Characterization of the species cross reactive anti-VEGF antibody SZH9.** A, SDS-PAGE of the homodimeric (scFv')$_2$ SZH9 antibody under reducing (left lane) and non-reducing conditions (right lane; middle lane, BSA control). The scFv monomer (approx. 29 kDa) and the dimer (approx. 58 kDa) are indicated by M and D. B, Western blot analysis of SZH9 binding to hVEGF$_{165}$, mVEGF$_{164}$ and cfVEGF. As negative controls BSA and VEGF-E were used. Proteins, 0.25 µg/lane were separated by SDS-PAGE on a 13% gel under non-reducing conditions using the SZH9 antibody as primary antibody. VEGF dimers (D) are indicated (approx. 43 kDa). The control antibody A1 did not produce a signal (data not shown). C, Binding curves of SZH9 to immobilized mVEGF$_{164}$,hVEGF$_{165}$ and cfVEGF. As negative controls BSA and VEGF-E were used. The control antibody A1 did not bind (data not shown).

Fig. s5, D-G

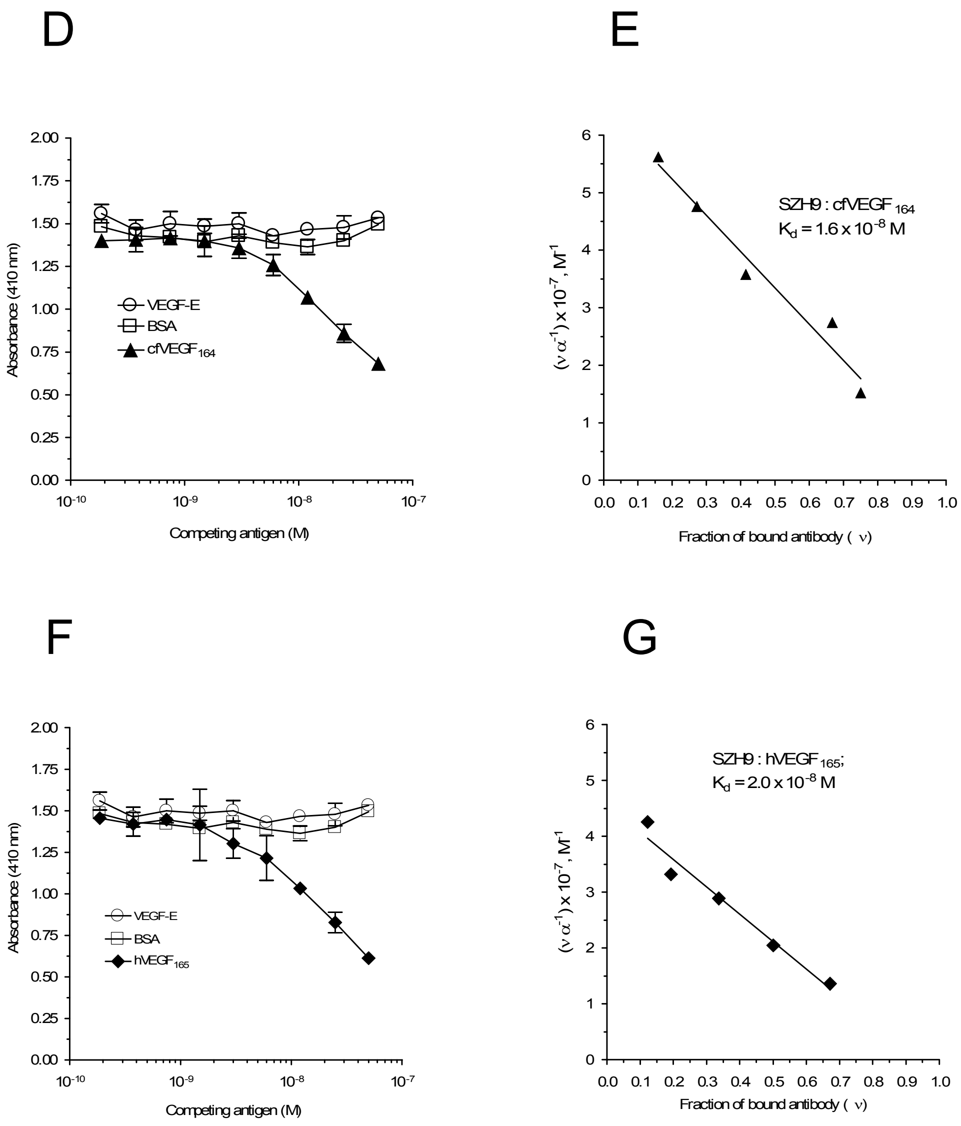

Fig. s5, D-G, **Competitive inhibition of SZH9 binding on ELISA plates pre-coated with VEGF in the presence of soluble dog (D, E) and human (F, G) VEGF**

Data are expressed as the mean ± SD (n = 3). The total concentration of SZH9 antibody was 10^{-9} M. Equilibrium was reached after 12 h at 4°C.

E, G Scatchard plots of binding of dog (E) and human (G) VEGF to SZH9. Binding parameters were calculated according to Friguet *et al*.(J. Immunol. Meth. 77, 305-319, 1985) where ν corresponds to the fraction of bound scFv and α to the concentration of free antigen at equilibrium. The total concentration of SZH9 was 10^{-9} M. All samples were analyzed at least in duplicate. Equilibrium was reached after 12 h of incubation at 4°C.

Fig. s6 A, B

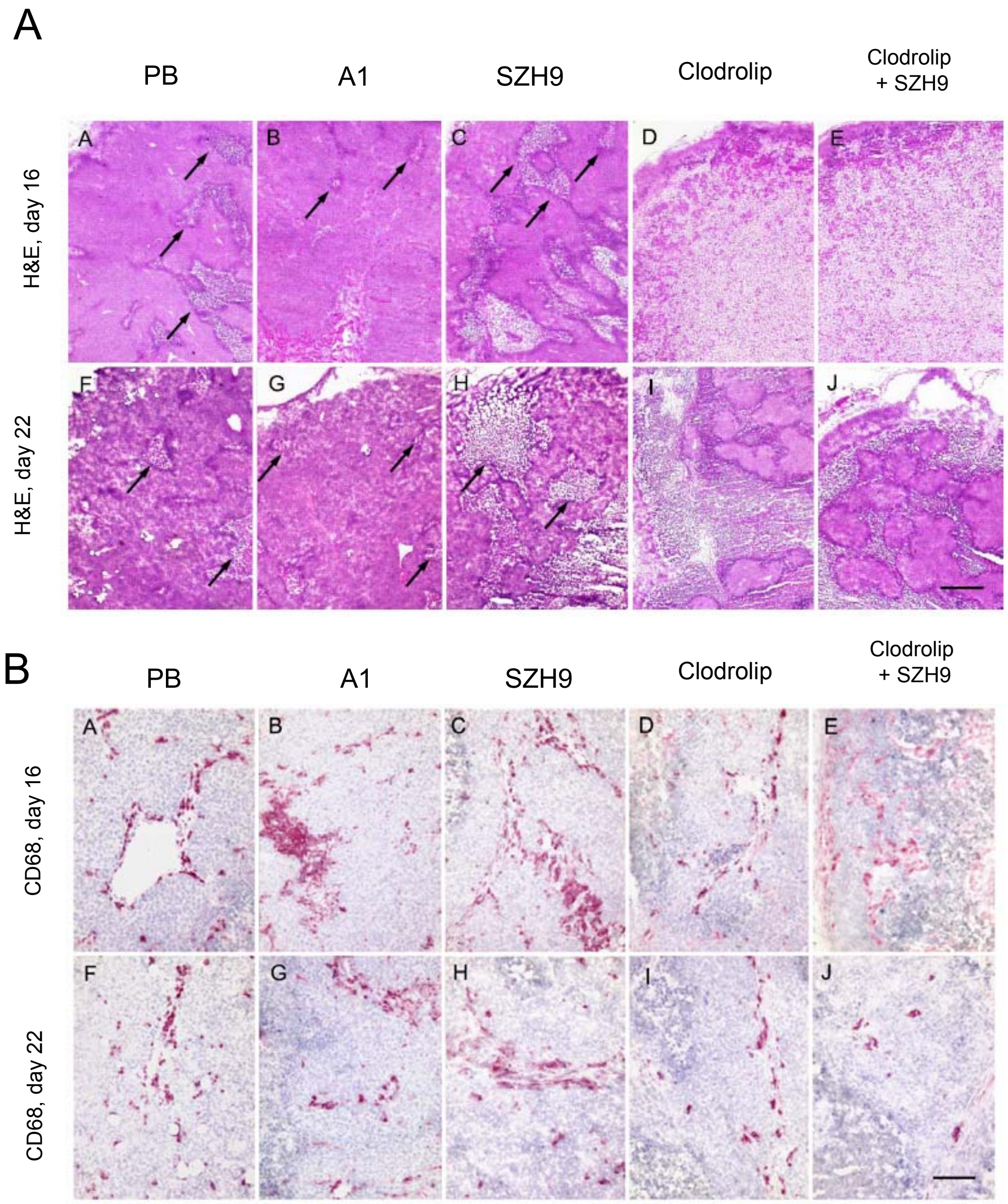

H&E staining and IHC of A673 tumors. A673 tumor sections from the treatment experiment shown in Fig. 3, C, D are shown. Stainings are from sections taken at days 16 and 22. A, H&E and B, CD68. ER-TR9 and LYVE-1 were negative in all sections (not shown, see Table 1). Arrows in A show necrotic areas. Bar = 200 μm.
